# Resorcinol in Dermatology: Reassessing a Legacy Topical Therapy for Contemporary Clinical Use

**DOI:** 10.1159/drm/ackag017

**Published:** 2026-09-08

**Authors:** Omar Raheel, Natalie Asemi, Aarushi Gulati, Victoria McGuirt, Maham Usmani, Steven R. Feldman

**Affiliations:** a Department of Dermatology, Wake Forest University School of Medicine, Winston-Salem, NC, USA; b Touro College of Osteopathic Medicine, Middletown, NY, USA; c A.T. Still University, School of Osteopathic Medicine, Mesa, AZ, USA; d Ohio State University College of Medicine, Columbus, OH, USA; e Department of Pathology, Wake Forest School of Medicine, Winston-Salem, NC, USA; f Department of Social Sciences & Health Policy, Wake Forest School of Medicine, Winston-Salem, NC, USA

**Keywords:** Resorcinol, Hidradenitis suppurativa, Acne, Hyperpigmentation, Keratolytic

## Abstract

**Background:**

Resorcinol is a phenolic topical agent with a long history in dermatology, but its role in contemporary practice has narrowed substantially. This review summarizes the mechanism, formulation considerations, clinical applications, safety profile, and future relevance of topical resorcinol.

**Summary:**

Resorcinol has keratolytic, antiseptic, antifungal, anti-inflammatory, and tyrosinase-inhibitory properties that support its use in acne, hidradenitis suppurativa, chemical peels, hyperkeratotic disorders, and pigmentary conditions. Despite historical use in acne, current evidence and guideline support are limited, and resorcinol remains largely absent from routine acne management. In contrast, topical resorcinol has a more defined role in mild to moderate hidradenitis suppurativa, particularly as a nonantibiotic option for localized inflammatory lesions. Safety concerns, especially thyroid toxicity, have contributed to its decline. However, reported systemic toxicity largely involved prolonged high-dose application to ulcerated or compromised skin, which differs from limited modern dermatologic use. Practical barriers, including lack of commercial availability at clinically relevant concentrations and dependence on compounding, also limit adoption.

**Key Messages:**

Resorcinol may be better viewed as an underused legacy compound rather than an obsolete therapy.

## Introduction

Resorcinol, also known as 1,3-dihydroxybenzene, is a phenolic drug that has been used in topical dermatological formulations for more than a century [1]. The chemical is a benzene ring with hydroxyl groups in the meta configuration [2]. It is a white, crystalline solid with a mild aroma and bitter-sweet taste [1]. Resorcinol is highly water soluble and hygroscopic, displaying a pale-red color in air and in light [1].

Resorcinol has multiple therapeutic properties that support its clinical use. At concentrations ≥10%, it acts as a keratolytic agent by disrupting hydrogen bonds within keratin [3]. It also exhibits antiseptic and antifungal activity [4]. Moreover, the fungicidal effect of resorcinol or related resorcinol compounds, such as phenylethylresorcinol, may reflect disruption of fungal cellular defenses [5]. In cosmetic applications, it serves as a coupler in oxidative hair dye formulations. Beyond clinical use, resorcinol currently has applications in manufacturing rubber products, wood adhesive resins, industry resins, UV stabilizers, or dyes [4].

Historically and currently in dermatology, resorcinol has been used in formulations to treat acne vulgaris, hidradenitis suppurative, eczema, psoriasis, and various other fungal and hyperkeratotic disorders [2]. However, resorcinol's use in conventional dermatological practice, such as in over-the-counter (OTC) acne products, is now limited [6]. This decline is largely associated with concerns regarding systemic toxicity and its effect on the thyroid gland. Resorcinol has been classified as an endocrine-disrupting chemical primarily targeting the thyroid function. It inhibits thyroid peroxidase, a key enzyme in thyroid hormone synthesis [7]. While resorcinol usage likely declined as a result of these effects, these concerns may be overstated. Reported effects were largely associated with extreme exposure scenarios, including high concentrations applied to ulcerated, compromised skin over prolonged periods [7]. In controlled human studies, topical resorcinol used on intact skin did not cause clinically significant changes in thyroid function [8]. Despite regulatory and historical concerns, resorcinol may have therapeutic value. This review summarizes the formulation and mechanism of resorcinol, its historical and current usage, safety profile, and potential future applications (Fig. 1).

**Fig. 1.**
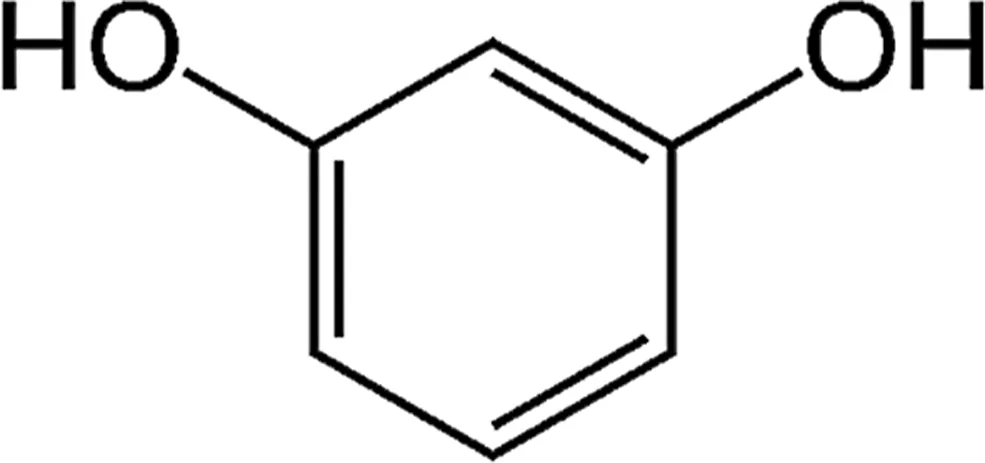
The chemical structure of resorcinol, an organic compound with the formula C_6_H_4_(OH)_2_.

## Methods

A literature review was conducted to evaluate the topical use of resorcinol in dermatology. A PubMed search was performed using the keyword “resorcinol,” combined with terms using the Boolean operator AND as well as describing topical application or formulation (topical, ointment, cream, peel, Jessner) and specific dermatologic conditions (acne, hidradenitis, keratosis, wart, leg ulcer). Studies were included if they examined the topical use of resorcinol in human subjects. Non-dermatologic applications and animal toxicology studies were excluded, except when discussed within regulatory evaluations relevant to human safety. Articles not published in English were excluded.

### Mechanism of Action and Formulation Considerations

Dating back to 1882, Paul Gerson Unna described resorcinol as a multifunctional dermatologic agent with keratolytic, antiseptic, and anti-inflammatory properties [9]. Mechanistically, resorcinol disrupts weak hydrogen bonds within keratin at the follicular infundibulum, thereby facilitating desquamation and relief of follicular occlusion [1, 3, 10, 11]. Resorcinol also acts as a selective inhibitor of tyrosinase and tyrosinase-related proteins, supporting its relevance in pigmentary disorders, while its keratolytic, antimicrobial, and anti-inflammatory properties support use in hyperkeratotic and inflammatory conditions [1, 3, 10, 11]. Structural modifications of resorcinol, including esterification with ethyl, glyceryl, and ascorbyl groups linked via alkyl chains, have yielded derivatives that inhibit tyrosinase activity and reduce melanin production at low concentrations without cytotoxicity [12]. Through tyrosinase inhibition, resorcinol reduces melanin production, providing a safe approach for managing pigmentary disorders [10, 11].

In addition to its keratolytic effects, resorcinol and its derivatives have antimicrobial activity, particularly antifungal effects mediated through disruption of fungal defense mechanisms and induction of cellular rupture [1, 5]. Phenylethylresorcinol, a derivative of resorcinol, completely inhibited *Trichophyton violaceum*, *Arthroderma cajetani*, and *Trichophyton mentagrophytes* at high tested concentrations [5]. These concentrations come from in vitro antifungal testing and should not be interpreted as clinically established topical treatment concentrations. Inhibition of *Trichophyton tonsurans* occurred at concentrations as low as 100 μg/mL, although the precise molecular mechanisms remain incompletely defined [5]. The anti-inflammatory mechanism remains under investigation, with conflicting data in the literature. While some secondary sources attribute resorcinol's anti-inflammatory effects to the promotion of prostaglandin E_2_ or leukotriene B_4_ [1, 3], ex vivo studies in human whole blood demonstrate that resorcinol potently suppresses prostaglandin E_2_ synthesis, indicating modulation of inflammatory responses through inhibition of prostanoid pathways rather than prostaglandin upregulation [13].

Resorcinol is available as an ointment, cream, or lotion in concentrations of 1–10%, though 15% has been used topically for HS [1]. FDA-approved combinations include resorcinol 2% with sulfur (3–8%) and resorcinol monoacetate 3% with sulfur (3–8%) at low dosages (1–2%). As a peeling agent, topical resorcinol is used at 5–15% in creams or pastes [1]. Resorcinol monoacetate provides a longer-lasting alternative by slowly releasing active resorcinol. Dosing is titrated to reach therapeutic concentrations while avoiding adverse effects, with final doses reaching at least 15% in cream form to minimize keratolysis. Maintenance therapy involves prophylactic application twice weekly [1]. Application to wounded skin should be avoided due to increased toxicity risk, as demonstrated by historical complications with high-concentration preparations for leg ulcers (up to 50%) [1].

Formulation, exposure conditions, occlusion, and skin integrity influence dermal absorption and tolerability. Regulatory evaluations conclude that dermal absorption is low under realistic use conditions, with systemic absorption generally ≤1–2% of the applied dose [14]. Notably, occlusion does not necessarily enhance penetration of hydrophilic compounds such as resorcinol [15], and aluminum tube packaging maintains stability at room temperature for at least 12 months [16, 17].

### Uses of Resorcinol

Current dermatological applications include OTC acne treatments, chemical peels (i.e., Jessner’s solution), compounded treatments for hidradenitis suppurativa (HS), hyperkeratotic disorders, and leg ulcers [1, 2]. Resorcinol’s anti-microbial, anti-inflammatory, and anti-keratolytic properties support therapeutic applications across multiple dermatologic conditions [18, 19]. Clinical use of resorcinol for acne in the USA is minimal. A retrospective study evaluating trends in non-first-line topical acne treatments from 2012 to 2016 found zinc and resorcinol scarcely used compared to first-line agents such as topical benzoyl peroxide and retinoids [20, 21]. For available OTC acne treatments, resorcinol is formulated in combination with sulfur or with sulfur and salicylic acid (35%) as it is considered ineffective as a monotherapy [20–22].

Despite limited clinical utilization data, resorcinol has clinical benefit as a monotherapy at varying concentrations in the management of mild-to-moderate HS. Traditionally, resorcinol has been used as a peeling agent at concentrations ranging from 5% to 15% for acne, most notably in Jessner's solution. Concentrations of 5–10% appear ineffective, while peeling emerged at a concentration of 15%. Unwanted side effects and systemic toxicities are rare and primarily reported in older literature [23]. In a prospective 1-year study of 15% topical resorcinol cream applied twice daily in 12 female patients with Hurley stage I or II HS, painful abscesses resolved in an average of 3.7 days (range 2–14) compared to 5 days to indefinite duration with placebo. Mean VAS pain scores decreased from 7.7 (range 7.7–8.2) at baseline to 3.4 (range 2.4–4.8) with treatment [23]. All patients experienced desquamation and 4 of 12 had discoloration, though moisturizer mitigated irritation [23]. Other concentration ranges of have also been evaluated. In an open, prospective, randomized trial topical resorcinol 10% was compared with topical clindamycin 1% in patients with Hurley stage I HS over 12 months. Patients in the resorcinol group applied a 10% oil-in-water cream formulation twice daily, while the clindamycin group used a commercially available product, and the third group served as the control [3]. At weeks 12 and 24, resorcinol had good tolerability with mild adverse effects and reduced total lesion count and improved quality of life compared to clindamycin [3]. Updated European guidelines recommend resorcinol therapy as an appropriate option for patients with mild-to-moderate HS [10]. Specifically, resorcinol may be used in Hurley stage I and mild stage II disease at a concentration of 15%, applied twice daily during flares and once daily as maintenance therapy for up to 16 weeks [10].

Resorcinol is a key component of chemical peels such as Jessner’s solution, which has been used for over 100 years to treat acne and hyperkeratotic lesions [24]. Jessner’s solution consists of 14% resorcinol, 14% salicylic acid, and 14% lactic acid dissolved in ethanol [25]. In a comparative study of 40 patients with mild-to-moderate acne, a 30% salicylic acid peel reduced Michaelsson acne scores and comedone counts by week 12 compared to Jessner's solution (salicylic acid group *p* < 0.05 vs. Jessner’s solution, *p* = 0.06); however, no significant differences were observed between groups for papule or pustule counts [24]. No cases of allergic reaction, persistent erythema, or post-treatment hypo- or hyperpigmentation were reported [24]. In another study, 36 patients with Fitzpatrick skin types IV–V were enrolled in a double-blinded, split-face, controlled trial comparing Jessner’s solution and salicylic acid peels administered every 2 weeks for three sessions [26]. Both treatments reduced inflammatory and non-inflammatory lesions, Michaelsson acne scores (*p* < 0.001), and post-acne hyperpigmentation. However, Jessner’s solution had faster improvement in hyperpigmentation at week 2 (*p* = 0.012) compared to salicylic acid, with comparable results by week 4 [26].

Other resorcinol derivatives such as isobutylamido-thiazolyl-resorcinol (ITR) have promising efficacy and safety for hyperpigmentation management in patients with skin of color, specifically Fitzpatrick skin types IV-VI, by inhibiting melanin production without common side effects [27]. In a blinded, randomized comparative trial in patients with Fitzpatrick types IV-V, ITR either alone or combined with hyaluronic acid improved melasma [28]. In Brazilian women with Fitzpatrick phototype V and acne-related post-inflammatory hyperpigmentation, ITR application twice daily for 12 weeks resulted in lighter treated sites compared to controls after 2 weeks, with continued improvement and significant post-inflammatory hyperpigmentation reductions by Mexameter measurements, expert grading, self-grading, and clinical photography at 12 weeks [11]. These findings represent resorcinol derivatives as reliable, gentle options for addressing hyperpigmentation in skin of color [27].

### Safety Profile and Pharmacokinetics

In cosmetic products, resorcinol is listed as 1,3-benzenediol and 1,3-dihydroxybenzene and is in hair dyes, lotions, and shampoos [29]. The 2022 Scientific Committee on Consumer Safety (SCCS) confirmed its safety up to 1.25% in oxidative hair dye formulations intended for hair and eyelashes, noting it does not act as an eye irritant [14, 30]. Acting as a moderate skin sensitizer, the frequency of contact sensitization to resorcinol in humans is low with cosmetic use [30, 31].

Resorcinol is readily absorbed through oral, dermal, and subcutaneous routes and metabolized as glucuronide conjugates, though conflicting evidence continues to fuel debate on its systemic safety [32]. Metabolism in the skin is slow and variable, with 5–77% of resorcinol remaining as parent compound after 24 h [14, 30]. Following systemic absorption, resorcinol is rapidly metabolized in hepatic cells with a half-life of 22–55 min and excreted primarily as monoglucuronide conjugate in urine within 24 h, with no accumulation in any organ or tissue, including the thyroid gland [14, 30]. In a controlled human study, 2% resorcinol twice daily (800 mg on 30% body surface area) was applied for 2 weeks to assess percutaneous absorption, blood levels, and urinary excretion. Resorcinol appeared safe for topical use in humans, with limitations owing to a sample size of three subjects, and further studies with larger cohorts are needed [8].

Systemic toxicity concerns center on thyroid suppression and hematologic effects. Based on human data, thyroid effects may occur following dermal exposure to ulcerated or compromised skin at resorcinol doses greater than 30 mg/kg per bodyweight per day (bw/day), with a thyroid effect threshold of 10 mg/kg bw/day established based on a threefold safety factor [7, 30]. Clinical case reports from patients undergoing resorcinol therapy for dermatological indications have documented thyroid side effects when copious amounts are applied to integrity-compromised skin for months to years, with effect levels greater than 34 mg/kg/day [7]. Ten human cases published between 1950 and 1977 reported severe clinical hypothyroidism following long-term exposure via cutaneous ointments for ulcer treatment, with effects induced by daily doses of 2–140 mg/kg/day for 3 months to 13 years that resolve upon cessation [7, 33]. The mechanism underlying thyroid disruption involves inhibition of thyroid peroxidase seen in vitro using porcine, human, and rat thyroid preparations [34]. Since resorcinol is rapidly cleared from plasma through urinary excretion, continuous exposure allowing sustained systemic release is required to produce thyroid effects [7, 30]. Intermittent or low-dose exposure does not cause hypothyroidism or other adverse health effects [14]. Epidemiological studies were unable to link four cases of hypothyroidism to occupational exposure, supporting that exposure at occupational concentrations is insufficient to cause thyroid abnormalities [7]. Classifying resorcinol as harmful without considering that systemic toxicity is influenced by skin integrity, concentration used, and body surface area exposed may lead to its therapeutic benefits not being realized at safe doses.

Evidence on resorcinol’s effects in infants, children, and pregnant patients remains limited and relies primarily on case reports. In infants and children, resorcinol should not be used on large body surface areas or wounds, as acute high doses can produce methemoglobinemia, particularly when applied to compromised skin. A case report documented methemoglobinemia in a neonate following use of benzocaine and resorcinol (Vagisil) cream for diaper rash, with a methemoglobin level of 35% on admission (normal 0.4–1.5%) [35]. In a case of accidental resorcinol poisoning, a pregnant woman at 30 weeks gestation ingested 50 g orally instead of glucose, resulting in unconsciousness, respiratory failure, tonic-clonic seizures, hypothermia, leukocytosis, elevated bilirubin, severe metabolic acidosis, and green-colored urine; the fetus died within 24 h after caesarean delivery, but the mother recovered with supportive management [36].

## Discussion

Resorcinol now occupies a narrow role in dermatology despite retaining properties that are relevant to several cutaneous disorders, including keratolysis, antiseptic activity, and utility in peeling formulations [1–3]. Its decline in routine practice appears to reflect a combination of factors rather than absence of therapeutic value, including limited commercial availability at clinically relevant strengths, dependence on compounding, competition from agents with larger modern evidence bases, and persistent concern regarding thyroid toxicity [21, 37].

In acne, contemporary use of resorcinol in the USA is minimal [6, 21]. The current over the counter acne monograph permits resorcinol only in specific sulfur-containing combinations and at restricted concentrations, which limits its practical market presence compared with benzoyl peroxide, retinoids, and other readily available agents [37]. The 2024 American Academy of Dermatology guideline also concluded that available evidence is insufficient to support a recommendation for topical resorcinol in acne, which likely further reduces interest in its routine use [21]. These regulatory and guideline realities likely help explain why resorcinol was not identified in the retrospective analysis of non-first line topical acne medications used in US office visits from 2012 to 2016 [6].

The current evidence for resorcinol is more compelling in HS than in acne [19, 23]. In this setting, its keratolytic effect is mechanistically relevant to follicular occlusion, and its topical use may offer a nonantibiotic option for recurrent inflammatory lesions [18]. European HS guidelines now include topical resorcinol 15% as an option for Hurley stage I and mild stage II disease, generally during flares and in some patients as maintenance therapy [10]. This is presently the clearest modern clinical niche for resorcinol in dermatology [3, 10].

Its possible antibiotic-sparing role also deserves attention [38]. In an era of increasing emphasis on antibiotic stewardship, a topical agent with keratolytic and antiseptic properties may be valuable in selected patients with HS, particularly when repeated use of topical or systemic antibiotics is undesirable [18, 38]. Resorcinol is unlikely to displace first line acne therapies, but in HS, it may fill a practical gap for localized inflammatory disease when a nonantibiotic topical option is preferred [3, 10].

Safety concerns remain central to the modern perception of resorcinol, especially with respect to thyroid function [39]. However, the historical reports that shaped this perception largely involved prolonged exposure, high concentrations, large treatment areas, and application to ulcerated or otherwise compromised skin, conditions that differ substantially from limited dermatologic use on intact skin or over small body surface areas [40–42]. This distinction is important because it limits direct comparison between older toxicity reports and current dermatologic practice patterns [39]. In one controlled human study, repeated topical application of 2% resorcinol to intact skin over 4 weeks did not identify clinically meaningful alterations in thyroid function, although the exposure conditions were modest and do not answer all contemporary pharmacokinetic questions [8]. Based on available data, the most severe systemic toxicities are associated with exposure scenarios that are not representative of typical modern dermatologic use [30, 39].

At the same time, regulatory caution has had lasting effects on clinician perception and product availability [43, 44]. European safety opinions continue to acknowledge endocrine-related concerns while also defining concentration limits considered acceptable in cosmetic products such as hair dyes and shampoos [14]. These assessments are important from a public health perspective, but they are not specific to limited, physician directed dermatologic use of compounded preparations over restricted body surface area [44]. As a result, the regulatory identity of resorcinol may appear broader and more concerning than its likely risk profile in carefully selected dermatologic settings [33, 43].

Another major barrier is formulation and access [10]. The concentrations most relevant to HS, particularly 10%–15%, are not available as standard branded dermatologic products in the USA and generally require compounding [16, 17]. This adds cost, inconvenience, and variability that are absent when prescribing commercially available alternatives [17]. Although compounded 15% resorcinol cream has been reported to maintain physicochemical and microbiological stability for 12 months in aluminum tubes at room temperature, the need for compounding remains a practical limitation to broader use [16, 17].

Resorcinol may still merit renewed study because it is a chemically simple and theoretically inexpensive small molecule with multiple relevant dermatologic properties [1, 2]. This may be particularly relevant in settings where cost constrains access to newer agents or where clinicians seek nonantibiotic topical therapies for recurrent disease [19]. Outside of HS, however, the evidence base remains limited, and broader adoption would require modern pharmacokinetic studies, standardized exposure thresholds, and comparative trials against established keratolytic and anti-inflammatory therapies such as urea, alpha hydroxy acids, beta hydroxy acids, and topical retinoids [39]. Additional work in skin of color populations would also be valuable, particularly because resorcinol derivatives have attracted interest for pigmentary disorders and post inflammatory hyperpigmentation with potentially favorable tolerability profiles [11, 27].

Overall, the available literature supports a selective rather than broad role for resorcinol in modern dermatology [21]. It is unlikely to return as a routine acne therapy, but it remains a plausible option in targeted settings, particularly mild to moderate HS, where its pharmacologic profile and existing clinical data are more aligned [3, 18]. Its decline appears to have been shaped not only by safety concerns, but also by market absence, compounding requirements, and the rise of more standardized alternatives with stronger modern guideline support [30, 37].

## Conclusion

Resorcinol has transitioned from a mainstream topical ingredient to a niche dermatologic therapy as a result of guideline evolution, regulatory caution, limited commercialization, and competition from newer agents with larger modern evidence bases. Current evidence does not support a substantial role for resorcinol in routine acne management, where more accessible and better studied therapies predominate. In contrast, the literature supports a more credible role for topical resorcinol in mild to moderate HS, where it may offer a useful nonantibiotic option for selected patients with localized disease.

The historical association between topical resorcinol and thyroid toxicity should remain part of any balanced discussion, but those reports largely arose in the context of prolonged high dose application to compromised skin and are not directly comparable to limited contemporary dermatologic use. On this basis, resorcinol may be better viewed as an underused legacy compound whose risks and benefits warrant reassessment under modern clinical and pharmacokinetic standards rather than as a therapy that is broadly unsuitable for dermatologic use.

## Conflict of Interest Statement

S.R. Feldman has received research, speaking, and/or consulting support from Eli Lilly and Company, GlaxoSmithKline/Stiefel, AbbVie, Janssen, Alovtech, vTv Therapeutics, Bristol-Myers Squibb, Samsung, Pfizer, Boehringer Ingelheim, Amgen, Dermavant, Arcutis, Novartis, Novan, UCB, Helsinn, Sun Pharma, Almirall, Galderma, Leo Pharma, Mylan, Celgene, Ortho Dermatology, Menlo, Merck & Co, Qurient, Forte, Arena, Biocon, Accordant, Argenx, Sanofi, Regeneron, the National Biological Corporation, Caremark, Teladoc, BMS, Ono, Micreos, Eurofins, Informa, Verrica, UpToDate, and the National Psoriasis Foundation. He is founder and part owner of Causa Research and holds stock in Sensal Health. All other authors have no relevant affiliations or financial involvement with any organization or entity with a financial interest in or financial conflict with the subject matter or materials discussed in the manuscript. This includes employment, consultancies, honoraria, stock ownership or options, expert testimony, grants, or patents received or pending, or royalties.

## Funding Sources

This paper was not funded. Funding was not provided for study design, data collection and analysis, decision to publish, or preparation of the manuscript.

## Author Contributions

All named authors meet the International Committee of Medical Journal Editors criteria for authorship, contributed substantially to the manuscript, approved the final version, and agree to be accountable for all aspects of the work. Omar Raheel was responsible for project design and conceptualization, manuscript writing, and final review. Natalie Asemi, Aarushi Gulati, Victoria McGuirt, and Maham Usmani contributed with literature review, manuscript drafting, and manuscript formatting. Dr. Steven Feldman contributed with project design, mentorship, manuscript editing, and review.
